# Limited Spectroscopy Data and Machine Learning for Detection of Zika Virus Infection in *Aedes aegypti* Mosquitoes

**DOI:** 10.3390/tropicalmed10110308

**Published:** 2025-10-29

**Authors:** Leonardo Reigoto, Rafael Maciel-de-Freitas, Maggy T. Sikulu-Lord, Gabriela A. Garcia, Gabriel Araujo, Amaro Lima

**Affiliations:** 1 Universidade Federal Fluminense, Niteroi 24220-900, RJ, Brazil; lreigoto@id.uff.br; 2 Laboratório de Mosquitos Transmissores de Hematozoários, Instituto Oswaldo Cruz, Rio de Janeiro 21040-360, RJ, Brazil; gabiazambuja@hotmail.com; 3 Department of Entomology and Arbovirology, Bernhard Nocht Institute for Tropical Medicine, 20359 Hamburg, Germany; 4 School of the Environment, Faculty of Science, The University of Queensland, Brisbane, QLD 4072, Australia; maggy.lord@uq.edu.au; 5 NIRSID Consortium, Brisbane, QLD 4072, Australia; 6 Program of Electrical Engineering, Federal Centre of Technological Education of Rio de Janeiro (CEFET/RJ), Campus Maracanã, Rio de Janeiro 20911-020, RJ, Brazil; gabriel.araujo@cefet-rj.br; 7 Program of Instrumentation and Applied Optics, Federal Centre of Technological Education of Rio de Janeiro (CEFET/RJ), Campus Maracanã, Rio de Janeiro 20911-020, RJ, Brazil; amaro.lima@cefet-rj.br

**Keywords:** Zika virus, machine learning, detection, arboviruses, classification, Support Vector Machine

## Abstract

This study presents a technique for categorizing *Aedes aegypti* mosquitoes infected with the Zika virus under laboratory conditions. Our approach involves the utilization of the near-infrared spectroscopy technique and machine learning algorithms. The model developed utilizes the absorption of light from 350 to 1000 nm. It integrates Linear Discriminant Analysis (LDA) of the signal’s windowed version to exploit non-linearities, along with Support Vector Machine (SVM) for classification purposes. Our proposed methodology can identify the presence of the Zika virus in intact mosquitoes with a balanced accuracy of 96% (row C2HT, average of columns TPR (%) and SPC (%)) when heads/thoraces of mosquitoes are scanned at 4, 7, and 10 days post virus infection. The model was 97.1% (10 DPI, row C2AB, column ACC (%)) accurate for mosquitoes that were used to test it, i.e., mosquitoes scanned 10-days post-infection and mosquitoes whose abdomens were scanned. Notable benefits include its cost-effectiveness and the capability for real-time predictions. This work also demonstrates the role played by different spectral wavelengths in predicting an infection in mosquitoes.

## 1. Introduction

Among the diverse group of arboviruses, certain pathogens such as dengue virus (DENV), Zika virus (ZIKV), and chikungunya virus (CHIKV) have emerged as significant global public health concerns due to their pathogenic potential and capacity to trigger explosive outbreaks in densely populated urban environments [1,2,3]. The primary vector responsible for the transmission of these arboviruses is *Aedes aegypti*, a mosquito species highly adapted to urban ecosystems. *Aedes aegypti* exhibits a strong anthropophilic behavior, preferentially feeding on human blood, and commonly oviposits in artificial water-holding containers located in or near human dwellings. Its limited dispersal range is typically restricted to a few hundred meters which further reinforces its close association with domestic and peridomestic habitats [4,5,6,7,8,9].

Given the rapid and unpredictable nature of arbovirus transmission, the effectiveness of vector control strategies hinges on the implementation of robust and timely entomological surveillance systems [10,11]. Surveillance programs based on the systematic sampling of immature and adult mosquito populations provide critical insights into vector abundance and spatial distribution, serving as the foundation for evidence-based public health decision-making [12,13,14,15,16]. However, conventional surveillance methods often suffer from limitations in spatial resolution and operational efficiency, necessitating refinement to meet contemporary demands [14,17,18].

To enhance vector control outcomes, mosquito surveillance data should be integrated into spatial and temporal analytical frameworks capable of generating dynamic risk maps [10,19,20,21]. These models can identify intra-urban transmission hotspots, enabling geographically targeted interventions that maximize resource allocation and operational impact. In this context, integrated vector management (IVM) strategies that align entomological surveillance with tailored control measures represent a promising paradigm for sustainable arbovirus control [22,23]. Ultimately, refined surveillance systems not only support epidemic preparedness and response but also facilitate continuous monitoring of disease trends, evaluation of intervention efficacy, and adaptive management of vector control programs. Such approaches empower local public health authorities to optimize control efforts and mitigate arboviral disease burden more effectively [24].

Recent advances in mathematical modeling and computational methods have significantly contributed to understanding and predicting mosquito-borne diseases such as dengue and Zika. Jose et al. (2024) conducted a comprehensive study on dengue incidence in Thailand, applying statistical and machine learning approaches—including exponential smoothing, polynomial fitting, and Random Forest models—to forecast outbreak dynamics and enhance public health preparedness [25]. In a complementary framework, Jose et al. (2023) developed a deterministic model describing the co-infection dynamics of Zika virus and dengue fever, highlighting through bifurcation and stability analyses the intricate interactions between human hosts and mosquito vectors [26]. From a biological control perspective, Joseph et al. (2023) proposed a fractional-order, density-dependent model to evaluate the dissemination of different Wolbachia strains in *Aedes aegypti* populations, identifying the wAlbB strain as the most effective in suppressing viral transmission [27]. Together, these studies demonstrate the potential of quantitative and data-driven approaches for understanding arboviral transmission and guiding vector control strategies. Building upon this foundation, the present study explores the integration of spectroscopy data and machine learning techniques as an innovative diagnostic approach for detecting Zika virus infection in *Aedes aegypti* mosquitoes, particularly under conditions of limited or sparse spectral information.

Zika virus (ZIKV), a member of the *Flaviviridae* family, was historically considered a geographically constrained and poorly characterized pathogen, primarily circulating in specific regions of Africa. However, in the early 2010s, ZIKV expanded its geographical range, culminating in a major outbreak in South America. In 2015, its association with severe neurological outcomes—such as congenital microcephaly in neonates and Guillain–Barré syndrome in adults—led the World Health Organization to designate it a Public Health Emergency of International Concern [28,29,30,31]. Although ZIKV transmission has declined globally following this epidemic, it remains an important model for studying the epidemiology and control of emerging and re-emerging mosquito-borne diseases. Effective surveillance of ZIKV, as with other arboviruses, requires surveillance tools that are not only sensitive and specific but also scalable, cost-effective, and operationally feasible for large-scale field deployment. Molecular diagnostic tools such as polymerase chain reaction (PCR) and quantitative PCR (qPCR) have long been the gold standard for detecting arboviral RNA in mosquito vectors [32]. Nevertheless, the logistical demands of these techniques—particularly their reliance on expensive reagents, skilled personnel, and time-consuming protocols—pose substantial barriers to widespread implementation in routine entomological surveillance. In response to these challenges, near-infrared spectroscopy (NIRS) has emerged as a promising alternative diagnostic modality. NIRS operates within a wavelength range of approximately 750–2500 nm and quantifies the absorption of light by specific chemical bonds (e.g., C–H, O–H, S–H, and N–H) within a biological sample [33,34]. The resulting spectral signatures reflect the molecular composition of the sample and can be analyzed to infer key biological parameters. This technique offers several operational advantages: it is non-destructive (preserving the sample for future analyses), reagent-free (minimizing costs), rapid (yielding results in seconds), and environmentally sustainable (generating no chemical waste) [35].

Researchers have previously utilized NIRS within the wavelength range of 700 to 2500 nm to identify the presence of the Zika virus in mosquitoes [36]. The obtained spectra were mean-centered and then subjected to classification through the Partial Least Squares (PLS) regression technique in GRAMS Plus/IQ software from Thermo Galactic. The outcomes of this method are presented in Table 1.

The primary issue with the approach outlined in [36] lies in its cost. Acquiring a near-infrared spectrograph can entail expenses running into the thousands of dollars. This study seeks to explore the feasibility of detecting the Zika virus in mosquitoes using a narrower band of wavelengths while leveraging advanced machine learning algorithms. Specifically, we apply Linear Discriminant Analysis (LDA) and Support Vector Machine (SVM) to the dataset from [36], focusing solely on wavelengths between 350 and 1000 nm. It is worth mentioning that other techniques, such as Principal Component Analysis (PCA) and Random Forest (RF), were also evaluated, but the most effective combination was the use of LDA and SVM. From a financial standpoint, a comprehensive near-infrared spectrometer (covering 350 to 2500 nm, which corresponds to the last part of ultraviolet, UV, the whole visible, VIS, and the whole near-infrared, NIR, spectra) typically carries a price tag around US $60, 000 (LabSpec 4i NIR spectrometer acquired and used in this work). In contrast, certain spectrometers capable of detecting light within the 350 to 1000 nm range can be found for around US $1500 (the model investigated was ATP1000−UV-VIS-NIR-5 from optosky.com). Electrochemical technology [37], with an estimated cost of tens of dollars, which identifies dengue and Zika viruses in solutions, cannot replace the spectrometer approach for this work, due to the fact that the conditions of applicability of the two technologies are quite different. For example, one requires a solution of the virus and the other requires the intact body of the mosquito. The main achievement of this work is to show that applying discriminative and classification techniques in a narrower spectral bandwidth could provide equivalent performance to the whole spectral bandwidth available in this work for identifying the Zika virus in intact mosquitoes, possibly reducing the implementation cost. The structure of this paper is as follows: Section 2 provides an overview of the dataset initially introduced in [36] and utilized in this study. Section 3 delves into the proposed methodology. Section 4 presents the obtained results and a subsequent discussion. Concluding remarks can be found in Section 5.

## 2. Dataset

The dataset utilized in this research was built and obtained from [36]. This dataset encompasses three key variables:

- The absorbance measurements at various wavelengths, ranging from 350 to 2500 nm;
- The infection status of the mosquito (infected or uninfected);
- The duration measured in days post infection (DPI).

**Figure 1:**
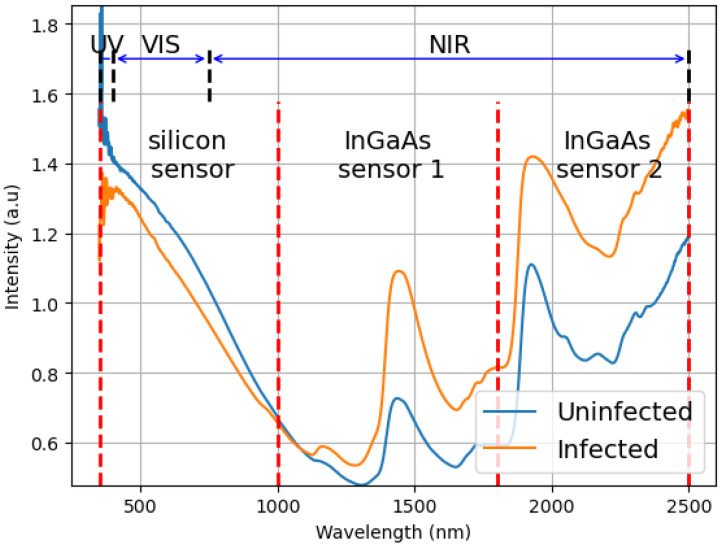
Example of the difference between the average spectrum of the mosquitoes infected and non-infected. This graph plots the result of the spectroscopy (intensity of light) versus the measured wavelengths (350 to 2500 nm). It also shows the ranges of the three sensors used in the equipment, silicon (350 to 1000 nm), and InGaAs (sensor 1: 1000 to 1800 nm, and sensor 2: 1800 to 2500 nm) sensors and how they are related to UV (350 to 400 nm), VIS (400 to 750 nm) and NIR (750 to 2500 nm).

In Figure 1, a comparison of absorbance versus wavelength is presented for two distinct samples. The blue line represents an uninfected mosquito, whereas the orange line denotes an infected mosquito (7 DPI). It also presents three sensor ranges used in the spectrometer, silicon (350 to 1000 nm) and InGaAs sensors (sensor 1: 1000 to 1800 nm, and sensor 2: 1800 to 2500 nm), along with the UV, VIS, and NIR limits.

In this experiment, we utilized female *Ae. aegypti* mosquitoes that were either 5 or 6 days old when they received their first blood meal. Briefly, an infective blood meal containing human blood from an anonymous donor and Zika virus was offered to half of the mosquitoes reared under standard conditions (27 ± 2 °C, 70 ± 5 relative humidity). The other half received ZIKV-uninfected blood from the same donor, plus C6/36 cell culture instead of virus. We established two distinct cohorts of mosquitoes, each collected and measured independently, with scans collected at 4, 7, and 10 days post infection (DPI). Prior to taking measurements, the mosquitoes were immobilised by placing them in a sealed jar with a cotton ball soaked in ethyl acetate for 1 min. Subsequently, a NIRS spectrometer was employed to analyze the absorbance across a range of wavelengths from 350 to 2500 nm, utilizing the LabSpec 4i NIR spectrometer from Malvern Panalytical, which features an internal 18.6 W light source.

For Cohort 1, spectra were collected from the head/thorax of the mosquitoes. For Cohort 2 two separate spectra were collected as follows: spectra of the combined head/thorax and spectra of the abdomen. Following these measurements, RT-qPCR tests were conducted on infected mosquitoes to confirm infection.

**Figure 2:**
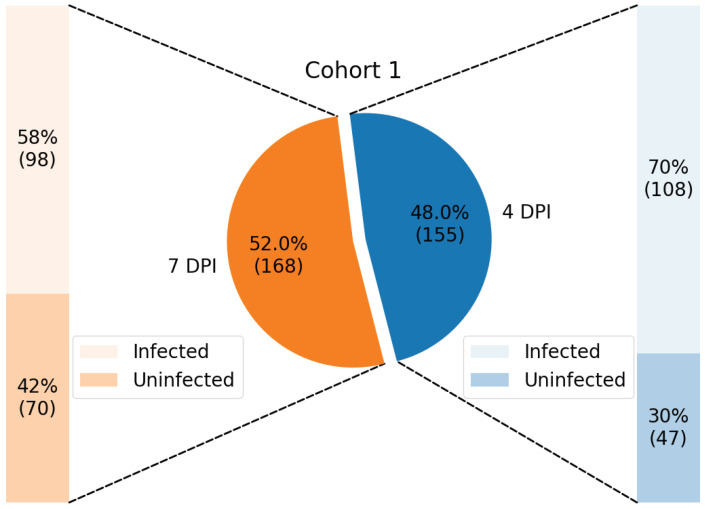
Distribution of samples from Cohort 1, training dataset, shown in percentage and with its respective number of samples enclosed in parenthesis.

**Figure 3:**
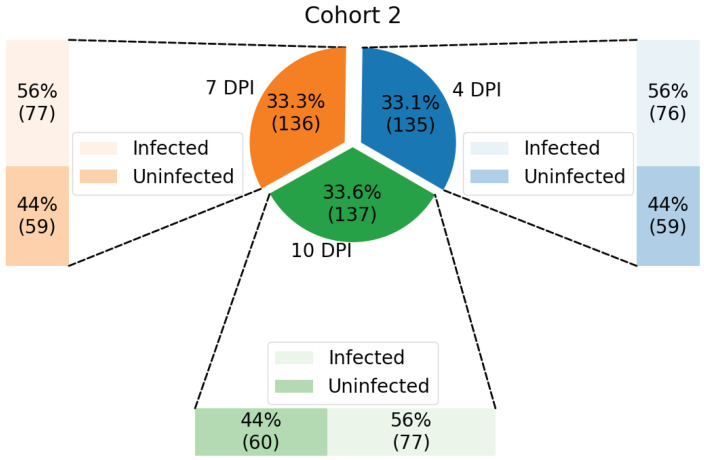
Distribution of samples from Cohort 2, test dataset, shown in percentage and with its respective number of samples enclosed in parenthesis.

Samples from each cohort were classified according to DPI (4, 7, and 10) and infection status (infected and not infected), resulting in up to six groups for each cohort, as illustrated in Figure 2 and Figure 3.

The data from Cohort 1 contains (a) 108 infected samples and 47 uninfected samples at 4 DPI; and (b) 98 infected samples and 70 uninfected samples at 7 DPI, making a total of 323 samples.

The data from Cohort 2 contains: (a) 76 infected samples and 59 uninfected samples at 4 DPI; (b) 77 infected samples and 59 uninfected samples at 7 DPI; and (c) 77 infected samples and 60 uninfected samples at 10 DPI, having 408 samples in total.

The spectrometer used combines three devices: (1) a silicon sensor that operates between 350 and 1000 nm; (2) an InGaAs sensor that operates between 1001 and 1800 nm; and (3) an InGaAs sensor that operates between 1801 and 2500 nm. Our model was designed using only the readings from the first device (the silicon sensor), as this wavelenght is most commonly found in low-cost equipment. However, a comprehensive comparison of efficiency among the other sensors data was also carried out.

## 3. Methodology

In this paper, we utilized samples from Cohort 1 to train our model and subsequently tested the model with data from Cohort 2, which can be considered unseen in the sense that it was not used in the training. This approach provides the benefit of having training and testing data that were collected and measured independently. Given that the preparation of mosquitoes and the associated measurements can be quite intricate, this separation of data aids in ensuring that our model can generalize effectively and predict the status of new samples rather than merely fitting to noise present in the data.

There are two issues concerning the remaining data: its limited number of samples and its high dimensionality. While excluding samples from Cohort 2 from our training data exacerbates the challenges posed by the scarcity of data, the benefits of this approach outweigh the drawbacks. It is essential to demonstrate that our model can generalize effectively. We employed the *k*-fold method (*k* = 10) to validate our model using samples from Cohort 1 without the necessity of removing them from our training dataset. Note that the validation data is part of the whole training data, Cohort 1. It is related to *k*-fold and used to find the hyperparameters associated to the most efficient *k*-fold model, which are used in the learning process of the whole training data generating a single model. The test data, Cohort 2, is related to the model evaluation.

The *k*-fold method involves partitioning the data into *k* distinct folds. We then trained our model *k* times, each time excluding one fold from the training data and utilizing it for model evaluation. The final evaluation metrics are derived from the average of all *k* models and the final model is a synthesis of all individual models. In classification tasks, the predicted class can be determined by the majority vote among all models. It is a common practice to assess a model using *k*-fold validation to find out the best hyperparameters associated the highest accurate *k*-fold model, which are subsequently used to create a new single model by training on all folds, i.e., on the whole training data, Cohort 2. If we obtain a sufficient quantity of data and folds to minimize noise from specific samples, it is anticipated, according to the theory of generalization [38] that our final model will closely resemble the amalgamation of the original *k* models.

### 3.1. Applying LDA

**Figure 4:**
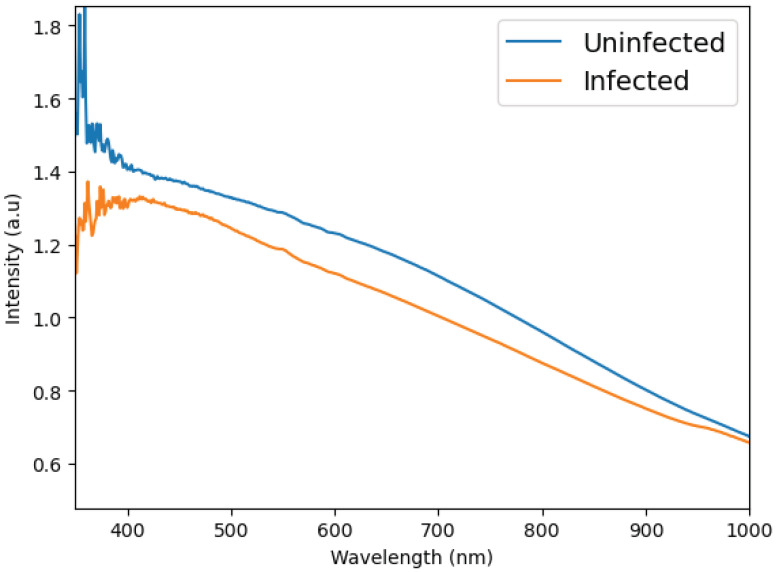
Example of the difference between the average spectrum of the mosquitoes infected and not-infected with ZIKV. This graph plots the result of the spectroscopy (intensity of light) versus the measured wavelengths (350 to 1000 nm).

To address the dimensionality issue, we eliminated data that contributes minimal information. Our dataset, which consists of absorbance measurements against wavelength, was expected to reveal some level of linearity since the data is being classified over a continuous range. This property allowed us to utilize Linear Discriminant Analysis (LDA) [39,40] as a feature extractor, effectively handling dimensionality challenges. LDA works by transforming the axes of our parameters into new dimensions that enhance class discrimination. Its primary objective is to identify a projection axis that maximizes the variance between classes while minimizing the variance within each class. By transforming the data in this way, it became possible to distinguish between classes using fewer parameters, which means we could confidently discard some of the original data elements. For a visual comparison, see Figure 4, which illustrates the spectral band from 350 to 1000 nm for both infected and non-infected mosquitoes.

**Figure 5:**
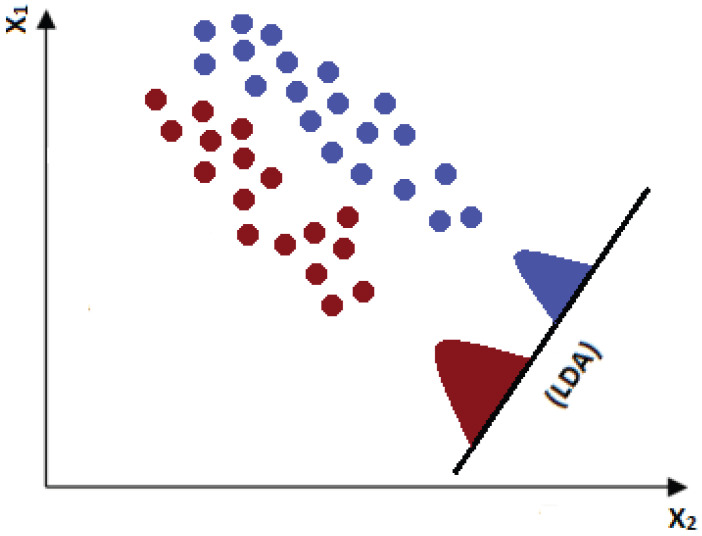
Illustrative example of a new axis generated by LDA.

The idea behind LDA is to transform our parameter axes in new ones that best discriminate our classes. The main focus of the LDA is to separate the classes by jointly maximizing the distance between their centers and minimizing the variance of each individually. This concept can be seen in Figure 5, where a new axis that indicates an optimization towards the discrimination between the classes is generated.

We employed the LDA algorithm to derive new features from our dataset. In classification scenarios involving *C* classes, the LDA algorithm yields C−1 axes that most effectively differentiate the classes. Given that the current problem only encompasses 2 classes (infected or not), the LDA algorithm produced merely 1 axis. Consequently, our model could only identify a threshold line along this axis and classify based on that threshold. A limitation of this method is its inability to address non-linearity, which could enhance the algorithm’s effectiveness. To overcome this limitation, we selected a reduced wavelength range of (350,1000] nm, corresponding to the *i*th intensity vector $x_{i}=\left[x\left(1\right),\ldots ,x\left(650\right)\right]$, and partitioned it into twenty-six non-overlapping windows, each with intervals of 25 wavelengths, resulting in ranges of (350,375], …, (975,1000] nm. We generated LDA models for each window’s associated data, denoted as ${\mathrm{LDA}}_{1},\ldots ,{\mathrm{LDA}}_{26}$, plus one more LDA model obtained from the entire interval, denoted as ${\mathrm{LDA}}_{\mathrm{all}}$, and applied the vector window to its corresponding LDA coordinates, thereby producing the projection of the vector window in LDA space. The twenty-seven projections were concatenated to form the feature vector, $\left[x_{1}^{\prime },\ldots ,x_{26}^{\prime },x_{\mathrm{all}}^{\prime }\right]$. This vector consists of an axis that optimally discriminates each vector window plus the entire interval. Each axis can still be utilized to classify each window plus the whole interval based on a threshold, but they can also indicate the reliability of these classifications through the distance from the threshold.

**Figure 6:**
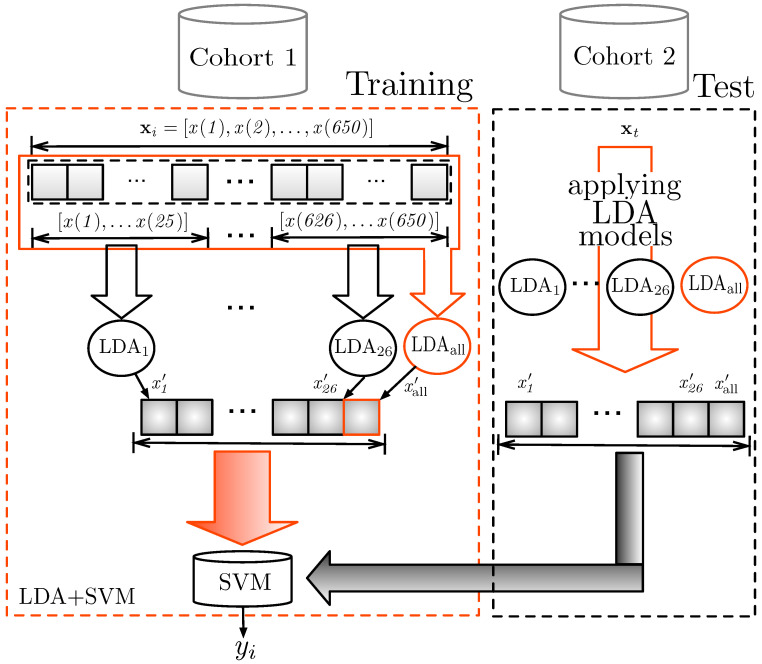
Block diagram of LDA + SVM approach and training and test setups.

After obtaining the feature vector by concatenating the projections from LDA, we utilized a Support Vector Machine (SVM) for data classification, which yields the output $y_{i}$ corresponding to $x_{i}$. The entire system, named as LDA + SVM, can be seen in Figure 6, showing the training and test sets are performed using Cohort 1 and Cohort 2 datasets, respectively. The LDA models for feature extraction used in test phase are the same generated during the training.

### 3.2. SVM for Classification

The Support Vector Machine (SVM) [41,42,43,44] is an exceptionally powerful and widely utilized algorithm. It has the capability to replicate the outcomes of more complex models while utilizing simpler ones. This characteristic enables the algorithm to manage a greater number of parameters with a reduced amount of data, demonstrating increased resilience against the *curse of dimensionality* [42] and *overfitting*.

The fundamental concept of SVM involves utilizing the hyperspace of input parameters to identify the optimal hyperplane that separates two classes. This hyperplane is designed to maximize the margin between the classes, which is determined by the support vectors. Support vectors are the data points that lie on the margin of the hyperplane. Furthermore, this margin classifier is capable of addressing non-linearly separable classes by transforming the data into a higher-dimensional space ϕ, which is practically executed through kernel functions, with the most prevalent being polynomial and radial basis kernels. We utilized the new vector generated from the LDA algorithms as input for a Support Vector Machine (SVM) with a polynomial kernel. By employing a non-linear kernel, our model gains additional complexity. The SVM aims to identify the hyperplane that best separates the data. As previously mentioned, our new vector represents the projection of each window along the axis that distinguishes it most effectively while also conveying the reliability of each discriminant. Essentially, the SVM aligns with the geometric structure of our data.

## 4. Results

The experimental results were generated by applying the proposed LDA + SVM system in the restricted wavelength range of 350 to 1000 nm for all subsets of the whole dataset (training and test), considering all different DPIs, and the composite performance using five evaluation metrics commonly applied to classification problems. A performance comparison between the three sensor ranges represented in the dataset plus the VIS spectrum was also evaluated. This second part of the results considered only the test set. The findings from the LDA + SVM approach considering the wavelength range from 350 to 1000 nm can be found in Table 2 and Table 3.

Table 2 presents the stratified results from the metrics True Positive Rate (TPR), also known as Recall and Sensitivity, Specificity (SPC), Precision (PRC), Accuracy (ACC), and F1-Score (F1S). Comparing the results displayed in Table 1 and Table 2, i.e., comparing two different models, it is observed that the proposed method attained a better TPR, which means a lower number of false negatives (FN) in almost all cases, except for C2HT at 10 DPI and C2AB at 7 and 10 DPI. However, considering the balanced accuracy $\left(\frac{\mathrm{TPR}+\mathrm{SPC}}{2}\right)$ in these three exceptions, the proposed method performed better, representing a certain balance between the percentages of true positives (TP) and true negatives (TN) within the actual classes. It is worth noting that a lower FN means the error of predicting infected mosquito as uninfected is reduced. Applying the same rationale, the SPC in the three exceptional cases is higher in the proposed method, meaning that the number of false positives (FPs) is lower, which represents a higher percentage of truly infected among all infected predictions. One important remark is that the model was trained without mosquitoes at 10 DPI, so this group of mosquitoes with higher balanced accuracy indicates a good generalized model, which can also be confirmed by observing the ACC metric for the C2HT dataset at 4 and 7 DPI, reaching 94.1% and 97.1%, respectively, and for the C2AB dataset at 4, 7, and 10 DPI, reaching 97.8%, 97.1%, and 97.1%, respectively. These results indicate generalization not only in unseen data but also in data without representation in training.

Table 2, row C2HT, column ACC (%) achieves 94.1% and 97.1% for 4 and 7 DPI, respectively. Also observing the same Table 2, row C2AB, column ACC (%) achieve 97.8% and 97.1% for 4 and 7 DPI, respectively. Furthermore, the same row and column for 10 DPI achieves 97.1%.

Table 3 presents the same results of Table 1 without DPI stratification, which is the statistics to be considered whenever this approach is applied in a field situation. For datasets C2HT and C2AB, no metric was lower than 92%, while the metrics TPR and F1S reached over 97%. Comparing the metrics in the same model, TPR values were higher than PRC with over 94%, indicating that the number of FP is higher than FN, and that the proposed model is more likely to find an infected mosquito than to miss it.

In order to analyze the proposed approach in different wavelength ranges, an experiment was devised applying the LDA + SVM method to the spectrum range concerning the original spectrometer silicon sensor from 350 to 1000 nm, InGaAs sensor 1 from 1000 to 1800 nm, and sensor 2 from 1800 to 2500 nm. In addition to that, the visible spectrum range, from 400 to 750 nm, was also added to the experiment. The results are presented in Table 4 for the test datasets C2HT and C2AB encompassing all the metrics (TPR, SPC, PRC, ACC, and F1S) with no DPI stratification. It can be noticed that the range originally chosen to develop the proposed model hit the higher performance in all metrics, probably because this spectrum range carries the most discriminative information to fulfill the task.

The second best range in terms of performance was the visible light, which showed higher predictive accuracy compared to InGaAs sensors 1 and 2 ranges, except for TPR in the range from 1800 to 2500 nm for both datasets. However, it was better than the InGaAs sensor 2 in both exceptions considering the balanced accuracy. Commercial instruments comprising the range of the silicon sensor and VIS are the cheapest available options with an average price of US $1500, while the InGaAs sensors 1 and 2 can cost up to US $32,000 (the models investigated were L/300-1100, L/400-740, N/900-1700, and N/900-2500 on simtrum.com). It is worth remembering that the proposed model, LDA + SVM model, was not trained with 10 DPI data nor abdomen measurements, i.e., the model was built without knowing any 10 DPI and abdomen data samples; however, it achieved performance above approximately 95% in these data strata.

## 5. Conclusions

The findings from this research indicate that it is feasible to detect Zika virus-infected mosquitoes using limited spectral data. This approach could potentially be executed with a more affordable device compared to the traditional Labspec spectrometer. As a result, it opens the door for the development of a budget-friendly IoT solution that can be utilized effectively in the field and improve surveillance of vector-borne diseases.

Reverse Transcription quantitative polymerase chain reaction (RT-qPCR) and standard PCR are the traditional technique for virus detection in mosquitoes [24,32,35]. However, it comes with several drawbacks: it is quite expensive, particularly for large-scale programs that require the analysis of thousands of samples, and it is time consuming and requires skilled personnel to operate. On the other hand, although it can be automated to provide results in real-time, the traditional NIR spectrometer currently in use for mosquito characterization (Labspec 4i) measures wavelengths from 350 to 2500 nm and requires more than US $60,000 as an overlay cost for a spectrometer. These drawbacks limit its applicability in many field settings. Our findings indicate smaller instruments with a smaller band width provide similar results as the traditional benchtop instruments. This is consistent with Takahashi and colleagues’ recent findings that demonstrated that a miniaturized spectrometer (NIRvascan) with wavelengths from 900–1700 nm is comparable to the LabSpec instrument for age grading and predicting mosquito blood feeding history [45]. Our findings suggest that a spectrometer with wavelengths from 350 to 1000 nm will provide similar results for detecting viruses in mosquitoes. Although cheaper spectrometers may have lower SNR, raising doubts about the noise robustness of the proposed system, the conclusion of [45] indicates equivalent discriminatory capacity of real data using cheaper equipment compared to more expensive ones.

The proposed LDA + SVM method demonstrated superior generalization in the classification of mosquitoes at 10 DPI when compared to the original approach examined in this study. The data for mosquitoes at 10 DPI and abdomen measurements are considered unseen, as they were not used during the training phase. The addition analysis of different ranges concluded that the VIS region on its own achieved slightly lower predictive accuracy than the range from 350 to 1000 nm, although the commercial equipment have similar costs. The key takeaway is that the proposed method achieves performance levels comparable to those reported in [36], while utilizing significantly less wavelength information.

Given the challenges associated with capturing and infecting mosquitoes, our training dataset is limited in sample size. To enhance the reliability of the statistical information, it is advisable to expand the dataset, preferably incorporating sampling infected and uninfected mosquitoes at different time points post-infection. This approach is expected to yield a more effective training model and potentially improve the accuracy of the system.

Further investigation into hyperparameter optimization and the removal of outliers is a logical next step, as the data has not been thoroughly explored. Future endeavors will also involve the development of a cost-effective IoT device to facilitate these readings and to implement our model in the field.

## Figures and Tables

**Table 1:** NIRS method [36], where C1TR stands for Cohort 1 train, C1VS for Cohort 1 validation (sorted), C2HT for Cohort 2 head/thorax, C2AB for Cohort 2 abdomen, TPR for True Positive Rate, SPC for specificity, and DPI for days post infection.

Dataset	4 DPI		7 DPI		10 DPI	
	**TPR (%)**	**SPC (%)**	**TPR (%)**	**SPC (%)**	**TPR (%)**	**SPC (%)**
C1TR	83.3	96.8	93.5	96.4	-	-
C1VS	100.0	94.1	100.0	100.0	-	-
C2HT	98.7	98.3	100.0	98.3	100.0	86.7
C2AB	98.7	85.0	96.2	80.0	97.4	68.3

**Table 2:** LDA + SVM (350 to 1000 nm) stratified in 4, 7, and 10 DPI, where C1TR stands for Cohort 1 train, C1VS for Cohort 1 *k*-fold validation, C2HT for Cohort 2 head/thorax, and C2AB for Cohort 2 abdomen, where TPR, SPC, PRC, ACC, F1S, are True Positive Rate, Specificity, Precision, Accuracy, F1-Score, respectively.

Dataset	TPR (%)	SPC (%)	PRC (%)	ACC (%)	F1S (%)
**4 DPI**					
C1TR	100.0	100.0	100.0	100.0	100.0
C1VS	100.0 ± 0.0	93.5 ± 10.3	96.5 ± 6.3	97.6 ± 4.2	98.1 ± 3.5
C2HT	100.0	86.4	90.5	94.1	95.0
C2AB	100.0	95.0	96.2	97.8	98.1
**7 DPI**					
C1TR	100.0	100.0	100.0	100.0	100.0
C1VS	100.0 ± 0.0	93.7 ± 9.6	94.8 ± 7.9	96.9 ± 4.8	97.2 ± 4.3
C2HT	100	93.2	95.1	97.1	97.5
C2AB	94.8	100	100	97.1	97.3
**10 DPI**					
C1TR	-	-	-	-	-
C1VS	-	-	-	-	-
C2HT	98.7	98.3	98.7	98.5	98.7
C2AB	94.8	100	100	97.1	97.3

**Table 3:** LDA + SVM (350 to 1000 nm) with no DPI stratification, where C1TR stands for Cohort 1 train, C1VS for Cohort 1 *k*-fold validation, C2HT for Cohort 2 head/thorax, and C2AB for Cohort 2 abdomen, where TPR, SPC, PRC, ACC, F1S, are True Positive Rate, Specificity, Precision, Accuracy, F1-Score, respectively.

No DPI Stratification (%)					
**Dataset**	**TPR (%)**	**SPC (%)**	**PRC (%)**	**ACC (%)**	**F1S (%)**
C1TR	100.0	100.0	100.0	100.0	100.0
C1VS	100.0 ± 0.0	91.8 ± 7.2	95.3 ± 4.3	96.9 ± 2.8	97.5 ± 2.2
C2HT	99.6	92.7	94.6	96.6	97.0
C2AB	98.3	96.7	97.4	97.6	97.8

**Table 4:** Comparing different wavelength ranges associated to the original spectrometer sensors (350–1000 nm, 1001–1800 nm, and 1801–2500 nm) and visible light (400–750 nm) without classifying mosquitoes into DPI for test datasets within Cohort 2 for head/thorax scans and within Cohort 2 for abdomen using TPR, SPC, PRC, ACC, and F1S metrics.

Range (nm)	TPR (%)	SPC (%)	PRC (%)	ACC (%)	F1S (%)
**Cohort 2 Head/Thorax**					
350–1000	99.6	92.7	94.6	96.6	97.0
1001–1800	90.4	41.6	66.7	69.1	76.8
1801–2500	98.3	36.0	66.5	71.1	79.3
400–750 (VIS)	93.5	88.2	91.1	91.2	92.3
**Cohort 2 Abdomen**					
350–1000	98.3	96.7	97.4	97.6	97.8
1001–1800	84.3	37.8	63.4	63.9	72.4
1801–2500	97.4	52.2	72.3	77.6	83.0
400–750 (VIS)	97.4	75.6	83.6	87.8	90.0

## Data Availability

The data set generated in this paper is available from the corresponding author on reasonable request.
